# The HYPERFlax trial for determining the anti-HYPERtensive effects of dietary flaxseed in newly diagnosed stage 1 hypertensive patients: study protocol for a randomized, double-blinded, controlled clinical trial

**DOI:** 10.1186/1745-6215-15-232

**Published:** 2014-06-18

**Authors:** Stephanie PB Caligiuri, Brian Penner, Grant N Pierce

**Affiliations:** 1 Canadian Centre for Agri-food Research in Health and Medicine (CCARM), St. Boniface Hospital Research Centre, 351 Taché Avenue, Winnipeg, MB R2H 2A6, Canada; 2 The Institute of Cardiovascular Science, St. Boniface Hospital Research Centre, 351 Taché Avenue, Winnipeg, MB R2H 2A6, Canada; 3 Department of Physiology, University of Manitoba, 745 Bannatyne Avenue, Winnipeg, MB R3E 0J9, Canada; 4 Department of Internal Medicine, University of Manitoba, 745 Bannatyne Avenue, Winnipeg, MB R3E 0J9, Canada

**Keywords:** Hypertension, Flaxseed, Blood pressure, Nutrition, Alpha linolenic acid

## Abstract

**Background:**

In 2013 the World Health Organization deemed hypertension as a global crisis as it is the leading risk factor attributed to global mortality. Therefore, there is a great need for effective alternative treatment strategies to combat a condition that affects 40% of adults worldwide. Recently, the FlaxPAD Trial observed a significant reduction in systolic and diastolic blood pressure in hypertensive patients with peripheral arterial disease that consumed 30 g of milled flaxseed per day for one year. However, these patients were already on anti-hypertensive medication. Therefore, there is a need to assess if dietary flaxseed can effectively reduce blood pressure in the absence of peripheral arterial disease and anti-hypertensive medication in newly diagnosed hypertensive patients.

**Methods/Design:**

The HYPERFlax Trial is a parallel, superiority, phase II/III, randomized, double-blinded, controlled clinical trial. St. Boniface Hospital and the Health Sciences Centre of Winnipeg, Canada, will recruit 100 participants newly diagnosed with stage 1 hypertension who have yet to be administered anti-hypertensive medication. Participants will be randomly allocated with a 1:1 ratio into a flaxseed or control group and provided food products to consume daily for six months. At baseline, two, four, and six months, participant assessments will include the primary outcome measure, averaged automated blood pressure, and secondary measures: 24-hour food recall, international physical activity questionnaire, anthropometrics, and blood and urine sampling for biochemical analysis. Plasma will be assessed for lipids, metabolomics profiling, and molecules that regulate vascular tone. Urine will be collected for metabolomics profiling. With an estimated dropout rate of 20%, the trial will have a power of 0.80 to detect differences between groups and across time, out of an effect size of 0.7 (SD) at an α level of 0.05.

**Discussion:**

This trial will determine if dietary flaxseed is efficacious over six months as an anti-hypertensive therapy in subjects newly diagnosed with hypertension. If flaxseed can effectively reduce blood pressure as a monotherapy, then flaxseed will provide individuals on a global basis with a cost-effective food-based strategy to control hypertension.

**Trial registration:**

NCT01952340, Registered 24 September 2013.

## Background

In 2013 the World Health Organization highlighted that high blood pressure is a global crisis [1]. Hypertension was identified as the number one risk factor attributable to most deaths worldwide and accounted for 16.5% of deaths [1]. Globally, in 2008, 40% of adults aged 25 years or older had hypertension [1]. Many individuals live with undiagnosed, untreated, or uncontrolled hypertension and this can lead to cerebrovascular disease, dementia, stroke, retinopathy, myocardial infarctions, heart failure, coronary artery disease, renal disease, vision impairment, and peripheral arterial disease [1]. Therefore, it is imperative to identify more effective and desirable treatment options to reduce the burden of hypertension globally.

Dietary interventions have been suggested as a preferential complimentary strategy to current pharmacological strategies to control blood pressure [2]. Flaxseed is one dietary intervention that has been used recently to reduce the risk of cardiovascular disease. Flaxseed has exhibited cardioprotective effects in animal models predisposed to cardiovascular disease by reducing atherogenicity [3-5], plasma cholesterol [4,6], plasma glucose [6], plasma trans fats [5], and blood pressure [7].

In humans, patients with peripheral arterial disease (75% hypertensive) were administered 30 g of milled flaxseed per day for six months [8]. Flaxseed consumption resulted in a large decrease in systolic (-10 mmHg) and diastolic blood pressure (-7 mmHg) that was statistically different from the control group [8]. Blood pressure was inversely associated with plasma concentrations of two flaxseed bioactives, alpha linolenic acid (ALA), and enterolignans [8].

Despite the significant anti-hypertensive effects demonstrated in the FlaxPAD trial, the results contained some important limitations. Firstly, participants of the FlaxPAD trial were already on anti-hypertensive medication yet still exhibited poorly controlled blood pressure [8]. Therefore it is not possible to conclude if flaxseed can reduce blood pressure as a monotherapy or if it is only effective in conjunction with anti-hypertensive medication. Secondly, it remained unclear if flaxseed could effectively reduce blood pressure in hypertensive patients without coexisting peripheral arterial disease. Therefore, despite evidence showing the potential of flaxseed as a potent cardioprotective functional food, the efficacy of flaxseed as an independent treatment to reduce blood pressure in newly diagnosed patients with primary hypertension has yet to be investigated.

The hypothesis to be tested in the present study is: dietary flaxseed will reduce blood pressure in patients with newly diagnosed hypertension and will reduce the need for anti-hypertensive medication. In order to test these hypotheses, a phase II, randomized, double-blinded, controlled clinical trial called HYPERFlax has been designed. The acronym for the HYPERFlax trial stands for the anti-HYPERtensive effects of dietary Flaxseed. Parameters such as blood pressure, plasma and urine metabolomics, plasma lipid profiling, and plasma vascular tone regulators will be analyzed to assess the efficacy and mechanisms of action of dietary flaxseed in hypertension management.

The aims of the HYPERFlax trial are to:

1. determine if consuming 30 g of milled flaxseed daily can effectively reduce blood pressure over six months in newly diagnosed stage 1 primary hypertensive participants;

2. determine if dietary flaxseed is efficacious as a monotherapy;

3. determine if flaxseed can reduce the need for anti-hypertensive medication; and

4. identify the mechanisms of action responsible for the potential anti-hypertensive effects of flaxseed.

## Methods/Design

### Design overview

The HyperFlax trial is a parallel, phase II, randomized, superiority, double-blinded, controlled study with an allocation ratio of 1:1. The participants, study coordinator (Stephanie Caligiuri), nurse, overseeing medical doctor (Dr Brian Penner), principal investigator (Grant Pierce), and researchers will be blinded to the group designation. One individual that dispenses the food products will be aware of the group designation. The trial sponsor is St Boniface Hospital. The trial has received approvals from the Health Canada Natural Health Products Directorate (#192282), the University of Manitoba Research Ethics Board (B2013:079), the St Boniface Hospital Research Review Committee (RRC/2013/1324), and the Health Sciences Department of Research (R12014:048). The trial is registered at clinicaltrials.gov (NCT01952340).

### Settings and participants

The trial will take place at the Health Sciences Centre Hospital and St Boniface Hospital in Winnipeg (Manitoba, Canada). The aim is to enroll 100 participants. Based on the previous FlaxPAD trial [8] the expected attrition rate is 20%, which still allows for a power of 0.80 to detect differences between groups and across time, out of an effect size of 0.7 (SD) at an α level of 0.05. Participants will be recruited through doctor referral or in response to public advertisements and public screening booths. The inclusion and exclusion criteria below fit the criteria of individuals who are not recommended to be administered anti-hypertensive medication immediately according to the Canadian Hypertension Education Program Guidelines [9]. These individuals do not have a systolic or diastolic blood pressure of more than 160/100 mmHg and do not have diabetes or macrovascular target organ damage. The exclusion criteria, therefore, allow a period of time for lifestyle intervention, including new therapeutic strategies like dietary flaxseed, in low-risk hypertensive individuals.

Inclusion criteria includes:

1. stage 1 essential hypertension (average automated systolic blood pressure of 135 to 160 mmHg or diastolic blood pressure of 85 to 100 mmHg) [9];

2. Newly diagnosed; defined as being clinically diagnosed within the last six months. This includes being diagnosed at the screening examination;

3. Male or female;

4. Untreated by medications for hypertension;

5. 18 to 85-years-old and able to provide informed consent;

6. Females who are not pregnant and not planning on becoming pregnant during the course of the trial. A pregnancy test will not be administered for the trial;

7. Subjects who are receiving anti-platelet therapy must be on a stable dose for three months prior to the study;

8. Subjects who are receiving lipid-lowering drugs must be on a stable dose for three months prior to the study;

9. Subjects must have access to freezer space in their residence to hold up to one month of the frozen food products associated with this study.

Exclusion criteria includes:

1. Patients with ischemic pain at rest in limbs, ulceration, or gangrene.

2. Patient has undergone percutaneous coronary angioplasty, or has had coronary bypass within the last six months.

3. Known secondary hypertension of any etiology.

4. Patients with confirmed and clinically significant renal or hepatic abnormalities (creatinine >0.130 mM or creatinine clearance <45 ml/min, AST (aspartate aminotransferase) two to three times the rate, ALT (alanine amino transferase) > more than two to three times the normal consentration) and/or electrolyte imbalance serum K (potassium) + <3.5 or >5.5 mM;

5. History of major bleeding;

6. Patients with diabetes mellitus, bowel disease (including Crohn’s disease, celiac disease, colitis, peptic ulcer disease, irritable bowel syndrome, and diverticulosis), or other diseases such as active systemic lupus erythematosus, cancer, or end-stage respiratory disease;

7. Patients with macrovascular target organ damage, including cerebrovascular disease, stroke, dementia, hypertensive retinopathy, left ventricular dysfunction, myocardial infarction, renal disease, and peripheral artery disease;

8. Patients with clinical evidence of heart failure or an estimated life expectancy of less than two years or with a high baseline cardiac risk (post-ischemic or diabetic cardiomyopathy with an ejection fraction of less than 40%, Canadian Cardiovascular Society Class 3 or 4 angina or a need for coronary revascularization procedures);

9. Subjects that are on supplements other that those prescribed by their clinician for the entire duration of the study. Please see point 10 below;

10. Subjects ingesting more than two servings of fish per week, taking omega-3 fatty acid supplements, and/or consuming milled flaxseed or flax oil on a regular basis (more than or equal to 1 tablespoon of milled flaxseed or 1 teaspoon of flax oil per week). If the participant is willing, a four-week washout period eliminating these supplements may be allowed before entry into the trial;

11. Patients who have participated in an investigational drug program in the proceeding 30 days or who are unable or unwilling to comply with the protocol;

12. Subjects with allergies to any ingredient in the study product or placebo (including gluten);

13. Patients who will undergo surgery or intend to move outside Winnipeg during the trial period.

### Consent

**Figure 1:**
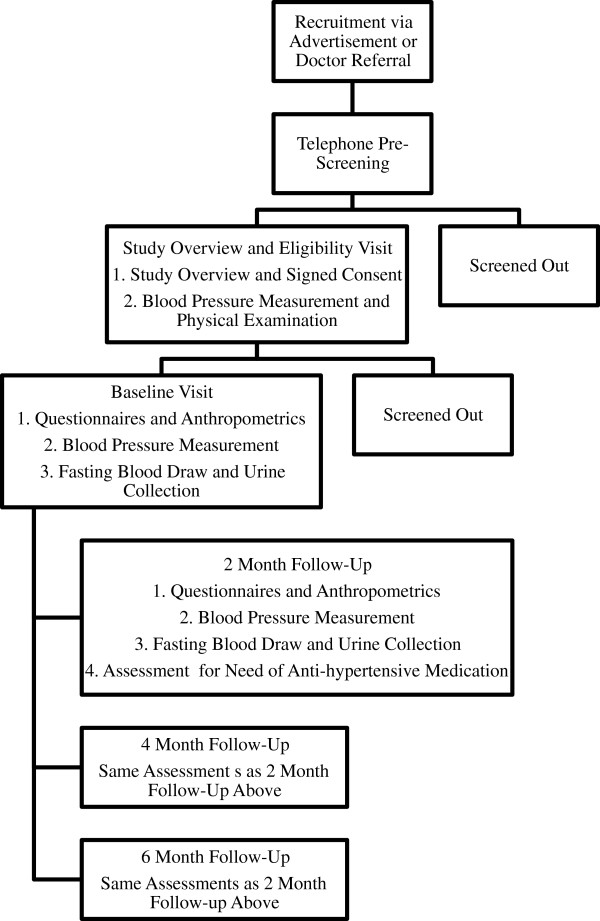
Study design overview.

Interested individuals will contact the study coordinator by telephone, upon which a pre-screening will be conducted to exclude participants based on the criteria above that can be determined over the phone. If the first criteria are met, a meeting will be scheduled to go through the consent and a secondary screening process will take place. Each participant will provide informed consent before the secondary screening process takes place. The study coordinator will again go through the exclusion and inclusion criteria to see if the participants are eligible for the study. Next, all study details will be outlined and the participants will be given a chance to ask any questions. If they consent to the study, the participant’s blood pressure will be measured six times within 10 to 15 minutes using a BPTru automated blood pressure machine BPTru, Coquitlam, BC, Canada to test if the participant has elevated blood pressure and meets the study criteria. The healthcare professional will also perform a brachial blood pressure measurement with a sphygmomanometer BPTru, Coquitlam, BC, Canada as is standard procedure at the doctors’ office. At this time, they will also see the lead medical doctor, Dr Brian Penner, at the hypertension clinic at the Health Sciences Centre for an examination to exclude any macrovascular target organ damage. If the participants meet the inclusion and exclusion criteria they will be notified by telephone of their acceptance into the trial if they still choose to participate. Figure 1 details the study overview and design.

### Randomization and interventions

Upon acceptance into the trial all participants will be provided information on lifestyle management of hypertension. These strategies include the dietary approaches to stop hypertension, limiting alcohol and salt intake, limiting or stopping cigarette smoking, increasing physical activity, and losing weight if the individual is overweight or obese. However, it is not mandatory to adopt these lifestyle changes to be eligible for the trial.

Immediately after the baseline assessments, subjects will be randomized to one of two possible groups by a non-restricted computer-generated randomization schedule. The allocation ratio will be 1:1. The study coordinator will enroll the participants and the individual in charge of food delivery will be in charge of participant allocation. The allocation order will be in sequential, opaque, sealed envelopes. At this point all those involved in the study will be blinded to the group allocation excluding the individual responsible for food delivery.

The participants will be provided either the control or flaxseed-containing food products such as muffins, bagels, snack bars, and milled seeds to consume once a day for six months. A variety of flavors will be provided to assure the participants do not tire of the foods. In addition, participants will be encouraged to remain in the study and be adherent by describing to them the importance of their contribution to identifying new treatment strategies for hypertension. The control food products will contain a combination of wheat, pecans, and/or mixed dietary oils to replace the flaxseed. This combination of ingredients for the control food product will and has allowed for the best concealment and ability for a blinded trial. The formulation and flavor profiling of the flaxseed food products has been previously published [10,11].

### Participant assessments

#### ***Averaged automated blood pressure***

The primary outcome measure is blood pressure. Blood pressure will be measured at a total of five visits: screening, baseline, two, four, and six months. A BPTru machine will be utilized to measure blood pressure six times within 10 to 15 minutes. The first reading will be discarded and the remaining five will be averaged. This measurement will take place in a quiet room while the participant is in a seated position and arm rested on an arm rest at heart level. A blood pressure reading with a sphygmomanometer and stethoscope will also be performed to ensure the congruency between this measurement and the automated measurement.

#### ***Need for anti-hypertensive medication***

One of the secondary outcome measures is the need for anti-hypertensive medication. In the first two months of the trial the participants will not be prescribed anti-hypertensive medication as this is the lifestyle intervention period. A meta-analysis published in 2008 concluded the administration of a placebo to patients with hypertension for four to eight weeks provided no objective reason against safety [12]. At two, four, and six months the participants will have a blood pressure follow-up. The participants will be assessed for the need for anti-hypertensive medication. If deemed necessary, they will be prescribed anti-hypertensive medication according to the Canadian Hypertension Education Program guidelines. If prescribed anti-hypertensive medication, the type and dose of anti-hypertensive medication will be recorded. If a follow-up visit is required sooner than the two-month interval, the patient will be asked to return to the clinic for more visits as standard of care.

#### ***Anthropometrics and questionnaires***

The secondary outcome measures also include anthropometrics, food intake, and physical activity. At baseline, the participants will be asked to fast (9 to 12 hour overnight fast) for blood and urine collection. The blood and urine will be analyzed utilizing techniques including metabolomics and lipid profiling. Biological specimens will be coded with an anonymous patient code and locked in a -80°C freezer. Height, weight, and waist circumference will be measured and body mass index (BMI) will be calculated to assess if the intervention may cause any changes in weight or waist circumference. They will also be asked to fill out a 24-hour food recall form with the study coordinator using the multiple-pass method as established by the United States Department of Agriculture [13] and the standardized international physical activity questionnaire (short) [14]. These two surveys are used to assess the participants’ typical food intake and physical activity habits. These assessments will be repeated at two, four, and six months to assess any changes in diet or physical activity over time, as detailed in Figure 1.

#### ***Biochemical analysis***

The secondary analysis will include the biochemical analysis of plasma or blood. Biochemical analysis also will be performed to determine potential anti-hypertensive mechanisms of action and adherence to intervention. Plasma samples from baseline, two, four, and six months will be analyzed for lipid profiles, metabolomics profile, and for any circulating plasma component that may influence blood pressure regulation. Plasma analysis of alpha-linolenic acid and enterolignans will act as adherence markers. Urine samples from baseline, two, four, and six months will also be analyzed for metabolomics profiling.

#### ***Statistical analysis***

All data will be stored without identifiable participant information in a password protected electronic document or locked cabinet. The final trial data set will be accessible only to researchers involved in the trial and any medical review board that requires access for safety purposes. In the likelihood of an unbalanced data set, absolute values will be analyzed with a mixed two-way repeated measures model with group and time as the between and within factor, respectively. A *post-hoc* comparison of the least squared means with a Tukey’s adjustment will be utilized to determine where the specific differences lie. Absolute change will also be calculated only for participants who provide clinical information and samples at all time points. Absolute change can be analyzed with one way analysis of variance (ANOVA) and the Kruskal-Wallis test, or multiple *t*-tests and the Mann Whitney U test with a false discovery rate correction. Multiple regression may be used to determine the influence of flaxseed on blood pressure while including factors such as age, BMI, gender, diet, or physical activity. Correlations will be utilized to determine relationships between biochemical markers and blood pressure. Subgroup analyses may also be performed, for example, the dichotomization of change in biochemical markers and observing subsequent change in blood pressure. All statistical tests will be set at a significance level of 0.05. Data will be published in peer-reviewed journals and discussed in public forums such as radio and television broadcasts.

### Reporting and evaluation of serious adverse events

No adverse effects are expected based upon published literature and our past experience with dietary flaxseed supplementation for one year [8]. Flaxseed has been granted Generally Recognized as Safe status by the Food and Drug Act 2009, USA and viewed as safe to consume for the general public [15]. Overall, toxicology and safety data in both human and animal trials have concluded flaxseed as safe to consume [16-23]. The food products provided to the participants will be prepared by companies that follow Good Manufacturing Practice of Health Canada. The perishable food products will be prepared, frozen, and stored frozen in a food warehouse. They will then be delivered frozen to the participants monthly.

Due to the ability of flaxseed to increase stool bulk and frequency of defecation, patients with a history of bowel obstruction, irritable bowel syndrome, or diverticular disease will be excluded. When large amounts of dietary fibre are consumed, gastrointestinal discomfort may occur. Results from our lab have indicated that the gastrointestinal discomfort and flatulence disappear within a few weeks once the participants have become accustomed to the fibre load. In addition, the gradual addition of flaxseed or wheat and wheat bran (control) throughout the first month allow the participants to become easily accustomed to the increase in fibre intake.

However, adverse events, as identified by the World Health Organization scale, will be followed up on if medically indicated, with relevant laboratory investigations under the direction of the study medical monitor. At this point, the medical board independent from the sponsor will be unblinded to the participant group allocation. Research staff will record the final outcome and the resolution date of the event wherever possible. If deemed necessary, the medical board will have the final say if the trial should be ended.

All serious adverse events (representing a significant health hazard to the participant) will be reviewed by the medical monitor within 24 hours of becoming aware of the events. The monitor will notify the Ethics Review Board within 7 to 14 days of the event. The University of Manitoba and St Boniface Hospital have research ethics boards that review all clinical trials for safety and ethical considerations. Health Canada, the University of Manitoba, or St Boniface Hospital may conduct audits at random to ensure adherence of guidelines. If any changes are required to be made to the protocol the above parties will be notified.

## Discussion

The HyperFlax trial will be the first study to investigate flaxseed as a therapeutic strategy for the reduction and management of blood pressure in patients newly diagnosed with hypertension who are yet to receive anti-hypertensive medication. Dietary flaxseed can reduce blood pressure in peripheral arterial disease patients already taking anti-hypertensive medication [8]. Therefore, the next logical step is to evaluate flaxseed as a monotherapy rather than in combination with anti-hypertensive medication, and without the added complication of peripheral arterial disease. The need for therapeutic strategies to reduce the prevalence and incidence of hypertension is necessary. Therefore, this investigation aims to provide essential knowledge on an alternative treatment strategy for hypertension management.

## Trial status

The trial has received approval from the Health Canada Natural Health Product Directorate, the University of Manitoba Research Ethics Board, the St Boniface Hospital Research Review Committee, and the Health Sciences Department of Research. Participant recruitment will start as early as April 2014.

## Abbreviations

ALA: Alpha-linolenic acid; BMI: Body Mass Index; FlaxPAD: Flaxseed for peripheral arterial disease; HYPERFlax: Anti-HYPERtensive effects of dietary Flaxseed; ANOVA: Analysis of Variance.

## Competing interests

The authors declare they have no competing interests.

## Authors’ contributions

SPBC, BP, and GNP designed the trial and are responsible for conducting the trial. SPBC and GNP wrote the manuscript. GNP has final responsibility for all content and all authors have approved the final manuscript.
